# Nichttuberkulöse Mykobakteriose nach Tätowierung durch *Mycobacterium chelonae*

**DOI:** 10.1007/s00105-023-05173-y

**Published:** 2023-06-21

**Authors:** K. Lange, S. Janßen, F. Dimmers, B. Homey, T. M. Jansen

**Affiliations:** Klinik für Dermatologie, Medizinische Fakultät, Heinrich-Heine-Universität, Universitätsklinikum Düsseldorf, Moorenstr. 5, 40225 Düsseldorf, Deutschland

**Keywords:** Tätowierung, Atypische Mykobakteriose, *Mycobacterium chelonae*, Tattoo-assoziierte Infektionen, Haut- und Weichteilinfektionen, Tattoo, Atypical Mycobateriosis, Mycobacterium chelonae, Tattoo-associated infections, Skin and soft tissue infections

## Abstract

Wir berichten über den Fall eines jungen gesunden Patienten, der sich mit juckenden Hautveränderungen im Bereich eines Tattoos des linken Handrückens in unserer Klinik vorstellte. Durch eine Biopsie und kulturelle Erregersicherung konnte eine Infektion mit *Mycobacterium chelonae* nachgewiesen werden, die wir mittels antibiotischer Therapie mit Azithromycin und Linezolid erfolgreich behandeln konnten. Unser Fall unterstreicht, dass neben allergischen Hautreaktionen auch Infektionen als Komplikation nach Tätowierung differenzialdiagnostisch in Betracht gezogen werden sollten.

## Abstract (en)

We report the case of a healthy young man who presented to our clinic with itchy skin lesions in the area of a tattoo on the back of the left hand. Bioptic and cultural confirmation of the pathogens led to the diagnosis of *Mycobacterium chelonae* infection. We initiated antibiotic therapy using azithromycin and linezolid with good response. Our case underlines that besides allergic skin reactions, infections as a complication after tattooing should also be included in the differential diagnosis.

## Anamnese

Ein 34-jähriger gesunder Patient stellte sich mit seit ca. 2 Wochen bestehenden Hautveränderungen des linken Handrückens im Bereich eines Farbtattoos in unserer Ambulanz vor. Anamnestisch seien diese 2 Wochen nach Stechen des Tattoos in einem professionellen Tattoostudio aufgetreten und mit leichtem Juckreiz sowie Brennen einhergehend gewesen. Der Patient hatte sich bereits mehrere Tattoos in der Vergangenheit stechen lassen, bislang ohne Komplikationen. Bis zur Vorstellung in domo erfolgte eine topische Therapie mit glukokortikosteroidhaltigen Externa ohne durchschlagenden Erfolg.

## Befund

**Figure 1:**
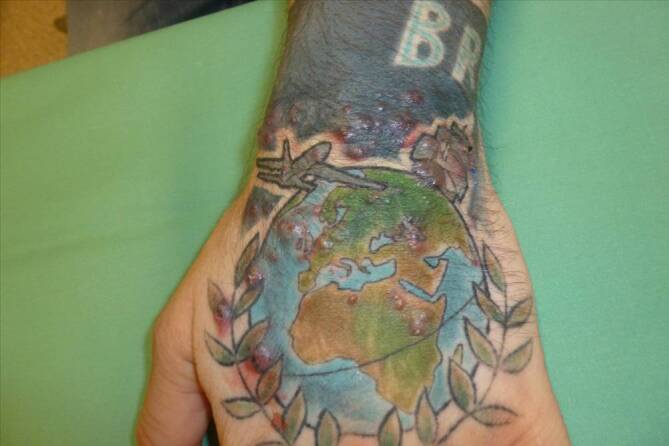

Klinisch imponierten bei Erstvorstellung am linken Handrücken im Bereich des tätowierten Areals multiple, zum Teil gruppiert stehende erythematöse bis zu 3 mm durchmessende Papeln und Pusteln (Abb. 1). Der Patient äußerte keine Infektsymptomatik.

## Diagnostik

**Figure 2:**
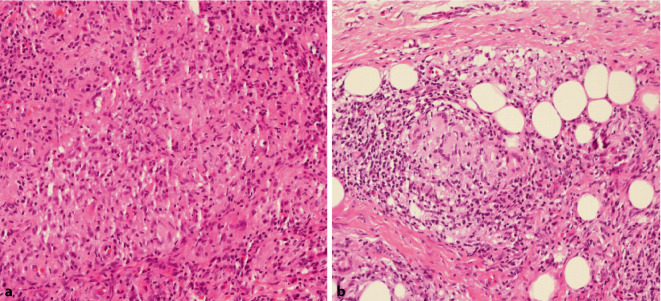

In einer durchgeführten Hautbiopsie zeigte sich eine epitheloidzellige granulomatöse Entzündung mit dem Nachweis von Fremdkörperriesenzellen (Abb. 2a, b). Mikroskopisch fehlte der Nachweis säurefester Stäbchen in der Ziehl-Neelsen-Färbung, in der Kultur zeigte sich jedoch ein Wachstum von *Mycobacterium chelonae.* Laborchemisch waren keine Auffälligkeiten erkennbar. Im extern durchgeführten Resistogramm zeigte sich eine Sensibilität gegenüber Azithromycin, Clarithromycin, Tobramycin und Linezolid.

## Therapie und Verlauf

**Figure 3:**
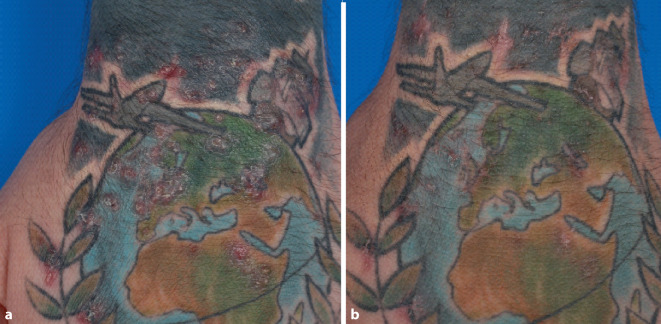

Initial leiteten wir eine Therapie mit Clarithromycin 500 mg 2‑mal täglich per os ein. Nach erfolgter Resistenzbestimmung und Vorstellung in der Mikrobiologie wurde die Therapie aufgrund häufiger Resistenzentwicklungen in der Monotherapie auf Azithromycin und Linezolid für 6 Monate umgestellt. Hierunter zeigten sich die Hautveränderungen regredient, nach erfolgter Therapie waren lediglich einzelne Hyperpigmentierungen im tätowierten Areal sichtbar (Abb. 3).

## Diskussion

Kutane Infektionen mit *Mycobacterium chelonae* können iatrogen durch medizinische und kosmetische Eingriffe erfolgen, wie beispielsweise nach Liposuktionen, Akupunktur oder Mesotherapie. Erst in den letzten 2 Jahrzehnten wurden Hautinfektionen durch nichttuberkulöse Mykobakterien mit Tätowierungen in Verbindung gebracht. Insbesondere in Frankreich, den USA, Spanien und Großbritannien wurden regionale Ausbrüche gehäuft beschrieben [2, 5, 7, 8], auch in Österreich wurde 1 Fall veröffentlicht [4]. In Deutschland wurde bis dato noch nicht über eine Tattoo-assoziierte Infektion mit *M. chelonae* berichtet.

Neben kontaminierten medizinischen Geräten oder Tinte kann eine weitere mögliche Infektionsquelle auch Leitungswasser, welches zur Verdünnung der Farben für beispielsweise Schattierungen genutzt wird, darstellen [1, 3]. *Mycobacterium chelonae *gehört zu den schnell wachsenden Erregern des *Mycobacterium-fortium*-Komplexes und stellt das am häufigsten beschriebene Mykobakterium in Assoziationen mit Tattoos dar. Die Hautveränderungen erscheinen meist 1 bis 3 Wochen nach Tätowierung, die Betroffenen sind in der Regel immunkompetent. Fieber oder Krankheitsgefühl wurden bislang nicht in Verbindung mit einer kutanen Infektion beobachtet. Die Sicherung der Diagnose erfolgt mittels Kultur oder PCR-Untersuchung aus einer nativen Hautbiopsie, die mit physiologischer Kochsalzlösung versetzt wird. Abstrichtupfer sind weniger geeignet. Sollte jedoch keine andere Probenentnahme möglich sein, sind auch diese in Transportmedien mit NaCl zu versenden [6]. Die sensitivste Methode zum Erregernachweis stellt die Kultur auf einem Löwenstein-Jensen-Agar dar, der einen lipidhaltigen Nährboden liefert. Ein Ergebnis liegt nach 8 bis 10 Wochen vor. Die antibiotische Therapie sollte mit Makroliden oder Fluorchinolonen erfolgen, in der Literatur wurde am häufigsten eine Therapie mit Clarithromycin für 3 bis 6 Monate beschrieben [3, 5]. Zur Resistenzbestimmung nichttuberkulöser Mykobakterien ist ein Speziallabor nötig. Aufgrund möglicher Resistenzbildungen auf Makrolide, welche v. a. durch Mutationen im *erm*-Gen entstehen können, ist häufig eine duale Therapie notwendig, sodass wir nach Rücksprache mit unseren Mikrobiologen eine antibiotische Behandlung mit Azithromycin und Linezolid initiierten. Dies wurde seitens des Patienten gut vertragen und führte zu einer sichtlichen Besserung des Hautbefundes.

## Fazit für die Praxis

Zusammenfassend zeigt dieser Fall, dass neben allergischen- und Fremdkörperreaktionen nach Tätowierung auch Infektionen als Komplikation differenzialdiagnostisch in Betracht gezogen werden sollten. Zudem können auch bei einem Auftreten von Hautveränderungen nach 2 bis 3 Wochen schnell wachsende Mykobakterien ursächlich sein.
